# Association of Morbid Obesity With Disability in Early Inflammatory Polyarthritis: Results From the Norfolk Arthritis Register

**DOI:** 10.1002/acr.21722

**Published:** 2013-01

**Authors:** J H Humphreys, S M M Verstappen, H Mirjafari, D Bunn, M Lunt, I N Bruce, D P M Symmons

**Affiliations:** 1 University of ManchesterManchester, UK; 2 University of East AngliaNorwich, UK

## Abstract

**Objective:**

Obesity has been associated with disease outcomes in inflammatory arthritis. This study aimed to investigate cross-sectionally the relationship between body mass index (BMI) and functional disability in a large inception cohort of patients with early inflammatory polyarthritis (IP).

**Methods:**

Patients age ≥16 years with ≥2 swollen joints for ≥4 weeks were recruited into the Norfolk Arthritis Register. At the initial assessment, clinical and demographic data were obtained, joints were examined, and height and weight were measured. Blood samples were taken to measure inflammatory markers and autoantibodies, and patients completed the Health Assessment Questionnaire (HAQ) to assess functional disability. Univariate and multivariate ordinal regression were used to examine the cross-sectional association between BMI and the HAQ. Multiple imputation using chained equations allowed inclusion of patients with missing variables.

**Results:**

A total of 1,246 patients were studied (median age 57 years). Of those patients, 782 patients (63%) were female and 303 (25%) were obese (BMI ≥30 kg/m^2^). Morbid obesity (BMI ≥35 kg/m^2^) was significantly associated with worse functional disability in the univariate and multivariate analysis with missing data imputed, adjusting for age, sex, symptom duration, smoking status, disease activity, autoantibodies, comorbidities, and treatment (multivariate odds ratio 1.87, 95% confidence interval 1.14–3.07).

**Conclusion:**

Morbid obesity in patients with early IP is associated with worse HAQ scores. This should be taken into account in patient management and when interpreting the HAQ in clinical practice.

## INTRODUCTION

Rheumatoid arthritis (RA) is a chronic inflammatory arthropathy that may be associated with significant disability through chronic inflammation and erosive joint destruction. The influence of body mass index (BMI) and body composition on disease outcomes, such as disability, in RA is unclear. Many RA patients are recognized to have altered body composition with a decrease in lean muscle mass despite stable overall body weight, a condition known as rheumatoid cachexia, and in underweight patients this is associated with poor prognosis (1). Less is known about prognosis in overweight or obese patients. Obesity (BMI ≥30 kg/m^2^) is rising in prevalence globally (2) and is a recognized cause of chronic disability (3). Obesity has the potential to affect functional disability in RA in a number of ways; for example, increasing BMI has been associated with more bodily pain and greater disablement for the same amount of pain (4), obese patients may be less able to undertake effective physical therapy due to their body habitus, and obese patients may have increased inflammatory burden due to metabolic activity of adipose tissue (1). Interestingly, in early RA patients, obesity has been found to have a protective effect on radiographic progression (5). However, obesity has also been associated with impaired health-related quality of life and disability in established RA (6, 7). It is not clear whether this is also the case in early disease.

Inflammatory polyarthritis (IP) is easily identifiable in a group of patients, of which RA patients constitute the major subset. In the early stages, RA can be difficult to distinguish from a wider group of inflammatory arthritides; therefore, it can be more useful to study IP patients to understand early disease processes. The aim of this study was to investigate the effect of obesity on functional disability in a large inception cohort of patients with early IP.

**Significance & Innovations**

- Body mass index (BMI) ≥35 kg/m^2^ is associated with worse Health Assessment Questionnaire (HAQ) scores in patients with early inflammatory arthritis.
- This association is independent of disease activity.
- With increasing obesity worldwide, BMI should be taken into consideration when interpreting HAQ scores in these patients.

## PATIENTS AND METHODS

### Setting

Patients in this study were participants in the Norfolk Arthritis Register (NOAR). Details of this register have been described elsewhere (8). Briefly, it aims to recruit all patients age >16 years with ≥2 swollen joints for at least 4 weeks in the former Norwich Health Authority area. Patients are referred to NOAR by their primary care physicians or hospital rheumatologists. All patients in this study were recruited into NOAR between the years 2000 and 2009 and had a disease duration of ≤2 years.

### Assessments

At inclusion into NOAR, patients were interviewed by a research nurse who administered a standard questionnaire, including details of symptom onset, comorbidities, and smoking status (current, ever, or never), as well as examining the joints (51 swollen and tender joint count) and measuring height and weight to calculate BMI (measured as kilograms per meter squared). A major comorbidity was one of a predefined list of 10 common diseases known to be associated with obesity and disability, including angina, myocardial infarction, stroke, and depression. Patients also completed the UK version of the Stanford Health Assessment Questionnaire (HAQ), a well-validated measure of functional disability in RA (9). Blood samples were taken to measure C-reactive protein (CRP) level and rheumatoid factor, and the remaining sera was stored frozen; this was later used to measure anti–cyclic citrullinated peptide antibody status. The Disease Activity Score in 28 joints (DAS28) was calculated based on 3 components (tender and swollen joint counts and CRP level) (10). NOAR is approved by the Norfolk Local Ethics Committee and all patients gave written consent.

### Statistical analysis

Patients were grouped according to their BMI (measured as kilograms per meter squared) into normal/underweight (BMI <25 kg/m^2^), overweight (25 < BMI < 30 kg/m^2^), class I obese (30 < BMI < 35 kg/m^2^), and class II and III obese (BMI ≥35 kg/m^2^, also known as morbid obesity) in line with the World Health Organization classification (2). HAQ scores were divided into tertiles. Missing data were imputed using chained equations (11). The relationship between BMI and HAQ score was analyzed using univariate ordinal regression to calculate odds ratios (ORs) and 95% confidence intervals (95% CIs). A multivariate model adjusted for age at symptom onset, sex, disease activity (as measured by the DAS28), smoking status, autoantibody status, number of major comorbidities, and whether the patient had already been started on disease-modifying antirheumatic drugs. Model analyses after each regression showed no significant difference between the proportionality of odds across the HAQ groups, confirming that this data set met the criteria for ordinal regression. All data were analyzed using STATA software, version 10.

## RESULTS

**Figure 1:**
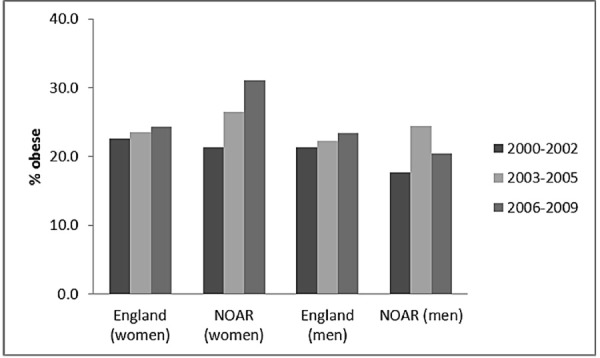
Prevalence of obesity over time by sex in the Norfolk Arthritis Register (NOAR).

A total of 1,246 patients with a disease duration of <2 years were recruited between 2000 and 2009. Of these patients, 782 (63%) were female, and the median (interquartile range) age at symptom onset was 57 years (46–69 years) (Table 1). A total of 636 patients (63%) fulfilled the 2010 American College of Rheumatology (ACR)/European League Against Rheumatism (EULAR) criteria for RA, and 517 patients (42%) fulfilled the 1987 ACR criteria for RA at baseline. A total of 303 patients (25%) in this cohort were obese (BMI ≥30 kg/m^2^); this comprised 93 men (21%) and 210 women (27%). Obesity was most prevalent among patients ages 35–44 years, comprising 16 men (42%) and 46 women (40%) in that age group. A total of 470 (60%) of women were overweight or obese (BMI ≥25 kg/m^2^); in men this figure was 293 (67%). Among women, the prevalence of obesity increased over the time of the study. Of the women who registered with NOAR in the year 2000, 23% (20 of 86 women) were obese; by 2008 that had increased to 31% (18 of 59 women). This increase appears to be greater than the overall rising prevalence among women in England, where national statistics show 21% of women were obese in 2000, increasing to 25% of women in 2008 (12) (Figure 1). In the age group similar to the median age in this cohort, 29% of women in England were obese in 2000, rising to 31% in 2008. A similar increase was not seen in men; this may be due to small sample size, as there were only 92 obese men in the cohort.

**Table 1:** Demographic and baseline disease characteristics*

	BMI <25 kg/m^2^	25≤ BMI <30 kg/m^2^	30≤ BMI <35 kg/m^2^	BMI ≥35 kg/m^2^	Total	Missing
Female	312 (68)	260 (57)	145 (67)	65 (75)	782 (63)	0
Age at symptom onset, median (IQR) years	58 (44–71)	59 (48–70)	55 (44–67)	49 (40–59)	57 (46–69)	83
Symptom duration, median (IQR) weeks	27 (18–46)	28 (15–47)	28 (19–51)	33 (20–53)	28 (17–48)	83
RF/anti-CCP antibody positive	238 (64)	231 (60)	100 (52)	48 (66)	617 (61)	227
2010 ACR/EULAR RA criteria positive	241 (63)	205 (56)	138 (72)	52 (72)	636 (63)	236
1987 ACR RA criteria positive	190 (41)	185 (40)	97 (45)	45 (51)	517 (42)	0
DAS28, median (IQR)	3.5 (2.6–4.5)	3.4 (2.4–4.4)	3.8 (2.9–4.9)	4.2 (3.3–5.2)	3.6 (2.6–4.6)	244
No. of comorbidities						0
None	316 (69)	295 (64)	118 (55)	37 (43)	766 (63)	–
1–2	84 (18)	102 (22)	50 (23)	27 (31)	263 (22)	–
>2	58 (13)	63 (14)	48 (22)	23 (26)	192 (16)	–
Smoking status						19
Never	196 (43)	177 (39)	67 (32)	43 (50)	483	–
Ever	141 (31)	187 (41)	101 (48)	34 (40)	463	–
Current	115 (25)	90 (20)	43 (20)	9 (10)	257	–
HAQ score, median (IQR)	0.75 (0.25–1.375)	0.75 (0.125–1.375)	0.875 (0.312–1.562)	1.375 (0.625–1.875)	0.875 (0.25–1.5)	0
HAQ score groups						
Group 1 (HAQ ≤0.375)	159 (35)	176 (38)	62 (29)	20 (23)	417 (34)	–
Group 2 (0.375 < HAQ ≤1.25)	170 (40)	154 (33)	80 (37)	22 (25)	326 (35)	–
Group 3 (HAQ 1.25)	129 (28)	130 (28)	74 (34)	45 (52)	378 (31)	–
Total	458 (38)	460 (38)	216 (18)	87 (7)	1,221	24†

* Values are the number (percentage of nonmissing data) unless indicated otherwise. BMI = body mass index; IQR = interquartile range; RF = rheumatoid factor; anti-CCP = anti–cyclic citrullinated peptide; ACR = American College of Rheumatology; EULAR = European League Against Rheumatism; RA = rheumatoid arthritis; DAS28 = Disease Activity Score in 28 joints; HAQ = Health Assessment Questionnaire.

† These patients had missing BMI data.

Almost all patients had complete data for BMI and HAQ scores (n = 1,221 and 1,246, respectively). However, there were some missing data in the covariates, with 496 patients (40%) having missing data on 1 or more of the covariates. In those patients with complete data (n = 825), BMI ≥35 kg/m^2^ was associated with higher HAQ score group in univariate and multivariate ordinal regression models (multivariate OR 2.43, 95% CI 1.34–4.39). In the imputed data set (n = 1,246), there was also a statistically significant univariate association between class II and III obesity (BMI ≥35 kg/m^2^) and higher HAQ score group. This association remained significant in the multivariate analysis (Table 2), adjusting for age, sex, disease duration, smoking status, DAS28, autoantibody profile, treatment, and the number of major comorbidities (OR 1.95, 95% CI 1.17–3.25).

**Table 2:** Ordinal regression models of association between BMI (independent variable) and HAQ score (dependent variable) after imputation of missing data*

	Univariate		Multivariate	
		
Independent variable	OR (95% CI)	FMI	OR (95% CI)	FMI
BMI (kg/m^2^)				
<25	Referent		Referent	
25≤ BMI <30	0.92 (0.72–1.17)	0.03	1.04 (0.79–1.35)	0.05
30≤ BMI <35	1.32 (0.98–1.77)	0.03	1.15 (0.82–1.61)	0.05
≥35	2.32 (1.49–3.61)†	0.02	1.94 (1.17–3.24)†	0.08
Age, years	–	–	1.03 (1.02–1.04)†	0.13
Female sex	–	–	1.88 (1.46–2.41)†	0.05
DAS28	–	–	2.44 (2.16–2.75)†	0.24
Current smoker (vs. nonsmoker)	–	–	1.69 (1.23–2.34)†	0.09
On treatment	–	–	1.53 (1.20–1.95)†	0.09

* BMI = body mass index; HAQ = Health Assessment Questionnaire; OR = odds ratio; 95% CI = 95% confidence interval; FMI = fraction of missing information; DAS28 = Disease Activity Score in 28 joints.

†Significant at *P* < 0.05.

## DISCUSSION

We have demonstrated that class II and III obesity, commonly known as morbid obesity, is significantly associated with HAQ disability in our cohort of patients with IP, independent of disease activity. The prevalence of obesity in this cohort was similar to the wider population reported in the annual Health Survey for England throughout the period of the study (12), with evidence of increasing prevalence over time. Among women in this cohort, the increase in prevalence over time is more marked compared to national statistics, which is in keeping with previous findings that obesity is a risk factor for the development of IP (13). The small number of obese men in the cohort may explain the absence of a similar pattern among men.

Functional disability is an important outcome in RA; in particular, when compared to other RA outcomes such as radiographic changes, it is important to patients as it relates directly to their activities of daily living. However, it is a complex construct that is influenced by many factors, of which BMI is just one. In this study, the proportion of variance in HAQ scores explained by all of the variables in the final model was 32%, and BMI explained the second highest share of that variance after disease activity. There remains, therefore, a considerable amount of residual confounding that we have not been able to adjust for and that should be investigated in future research.

To our knowledge, this is the first study to investigate the influence of obesity on disability in early IP. Of interest, obesity appears to influence radiographic damage in early RA in the opposite direction to HAQ score, with previous studies showing that obesity protects against erosive disease (5). This suggests that the relationship between obesity and disability may be independent of the disease itself, i.e., obesity adds further functional disability to the already disabled RA or IP patient. There is good evidence in the general population that obesity is associated with significant disability as measured by HAQ scores (3) and other quality of life measures (4). It is possible, in the previous studies assessing radiographic damage, that a subset of patients were misclassified as having RA when in fact they had another joint disease such as osteoarthritis (OA). This misclassification can occur in all studies of patients with inflammatory arthritis and is more likely to occur in obese patients due to the presence of chronic low-grade inflammation (1) and difficulties in accurately detecting synovitis in larger patients. In addition, OA is common in overweight and obese patients (4). If, indeed, a proportion of these obese patients had OA, they would not be expected to develop erosive changes on radiographs and obesity might appear to be protective. Unfortunately, we were not able to investigate this as baseline radiographic data were not collected at inclusion to NOAR.

It has been hypothesized that the increased adiposity of obesity might influence disease activity and, therefore, outcomes such as functional disability. This may be because obesity has been associated with chronic inflammation (1); adipose tissue is metabolically active, secreting a range of adipocytokines that are both pro- and antiinflammatory in nature (1) and that therefore have the potential to modulate disease activity. Supporting this hypothesis, some studies have identified an association between obesity and increased disease activity. Indeed, in this cohort there was an increasing DAS28 score with increasing BMI (Table 1). In a cross-sectional study of RA patients with more than 3 years disease duration, Stavropoulos-Kalinoglou et al found that high BMI was associated with increased erythrocyte sedimentation rate, an inflammatory marker frequently elevated in active disease (7). Further, the Quantitative Standard Monitoring of Patients with Rheumatoid Arthritis (QUEST-RA) Study found an association in women between BMI and disease severity as measured by high DAS28 (14). However, in this study the association between morbid obesity and disability was independent of disease activity.

Another possible explanation for the association between BMI and functional disability is reverse causation. This would occur if patients, as a result of more severe disease, are less able to undertake physical activities and exercise and as a result are overweight or obese. The effect should become more marked with longer disease duration. This was also investigated in the QUEST-RA Study, a multinational cross-sectional study of prevalent RA cases (14). They did not find good evidence for reverse causation in their cohort, as patients with high disease activity showed no correlation between disease duration and increasing BMI. Additionally, in our cohort this is less likely to be the case because of the short disease duration in the majority of patients.

There are limitations to this study. A significant proportion of patients had missing data, mostly relating to covariates. To account for differences between patients with missing and nonmissing data, we used multiple imputation using chained equations, a well-validated technique that allows inclusion of as much information in the final model as possible (11).

We have used BMI to identify obese patients, which is a fairly crude measure of body composition. Other tools have been suggested that may be more appropriate in RA patients who may have loss of lean muscle mass despite normal or stable body weight (1), such as hip to waist ratio, bioimpedence, or whole-body magnetic resonance imaging.

Another limitation is that our cohort is a group of patients with IP rather than exclusively patients with RA. However, 63% of this cohort satisfied the 2010 ACR/EULAR classification criteria for RA, and we have shown previously that more than 75% of patients with IP go on to meet the 1987 classification criteria for RA within 5 years (15). The strength of this type of cohort is that it ensures complete capture of all patients who may develop RA.

In conclusion, we have shown that morbid obesity is independently associated with HAQ disability in patients with IP. This result needs to be verified in other early arthritis cohorts; nevertheless, the role of obesity should be taken into consideration when interpreting HAQ scores in clinical practice. Importantly, these patients may need greater input from the multidisciplinary team to help them manage their limitations in activities of daily living.
